# Burrows on Cerebral Circulation

**Published:** 1848

**Authors:** 


### ARTICLE II.

*On the Disorders of the Cerebral Circulation, and on the Connection between Affections of the Brain and Diseases of the Heart.* By GEO. BURROWS, M.D., Lecturer on the Principles and Practice of Medicine in St. Bartholomew's Hospital, etc., etc. With colored plates. Lea & Blanchard: 1848.

The first part of this work is devoted to the cerebral circulation, and especially to contesting the doctrine sustained by Abercrombie, that the cranium is a sphere and contains, under all circumstances, the same quantity of blood

This doctrine, adverse to the current views of pathology as indicating that sanguine congestion plays a less important part in cerebral than in other diseases, is sufficiently disproved by daily observations of post mortem appearances, but the author of this work sets it at rest by experiments on animals. The brains of animals strangled and those bled to death differ greatly in appearances and in the amount of blood they contain.

The whole subject of pressure and its consequences is fully and judiciously treated, and another less important but deserving of more attention than it has received—viz., the want of pressure and its influence on the nervous functions—is dwelt upon to some extent.

The chapters upon the connection between diseases of the heart and those of the brain sustain the received views of the effects of hypertrophy of the heart and contraction of the aortic orifice in producing congestion and *ramollissement* of the brain.

There are also some interesting cases of pericarditis similating inflammation of the brain, an obscure and difficult point of pathology.

It is a work calculated to be of practical benefit, and we cordially recommend it to the profession. D. B.

